# Suspensory Device Fixation of Lisfranc Injuries in a Southeast Asian Urban Population: Patient-Reported Functional Outcomes

**DOI:** 10.7759/cureus.46629

**Published:** 2023-10-07

**Authors:** Yong-Yao Tan, Yi-Mei Low, Raj Kumar Socklingam, SBM Darshana Chandrakumara, Charles Kon

**Affiliations:** 1 Orthopaedic Surgery, Changi General Hospital, Singapore, SGP; 2 Orthopaedics, Changi General Hospital, Singapore, SGP

**Keywords:** sport injury, trauma, suture button, suspensory fixation, foot, orthopaedic, lisfranc

## Abstract

Introduction

Open reduction internal fixation (ORIF) and primary arthrodesis are two conventional options for the treatment of Lisfranc injuries. However, they are associated with implant-related complications. An alternative suspensory device construct using interosseous nonabsorbable sutures with endobuttons has been described with satisfactory results. This study aims to explore functional outcomes after suture button fixation of Lisfranc injuries in a Southeast Asian population.

Methods

This was a single-surgeon retrospective study of patients with Lisfranc injuries treated surgically using a suture button fixation technique between 2017 and 2019. Data collected included demographic information, pre-injury levels of activity, nature of injury, and type of surgery performed. The minimum postoperative follow-up was one year. The Foot and Ankle Outcome Score (FAOS) and Foot and Ankle Ability Measure (FAAM) were used to evaluate patient-reported outcomes. Scores were reported in percentage (%) with median and interquartile range.

Results

Twenty-nine patients with a mean age of 29 years (21-76) were recruited. Sixteen underwent suture button fixation only (SB), and 13 underwent suture button fixation with intercuneiform screw fixation and plating (SBM). The median scores for the FAOS and FAAM questionnaires were at least 80% in all domains. Twenty-eight patients (97%) were able to return to pre-injury activity level, 27 patients (93%) were able to return to sports. Only one patient was not satisfied with the outcomes of surgery. No patients had post-traumatic arthritis or hardware failure necessitating implant removal at the final follow-up.

Conclusion

This study has demonstrated that treatment of Lisfranc injuries with a suspensory device construct resulted in good outcomes with 97% of patients being able to return to pre-injury activity levels, and 93% of patients being able to return to sports. It may not be necessary to perform primary arthrodesis in uncomplicated Lisfranc injuries. This technique is also advantageous as implant removal is not routinely required due to the design and biomechanical properties of suspensory devices.

## Introduction

Injuries at the tarsometatarsal (TMT) joint are becoming increasingly common, with recent literature reporting an incidence of 14/100,000 person-years. This is more than eight times that reported by older studies [1]. The true incidence is likely to be higher as more than 20% of the estimated 55,000 Lisfranc injuries annually are missed on initial assessment. If not managed appropriately, patients with Lisfranc injuries may develop loss of transverse arch, widening of the first webspace, midfoot instability, and post-traumatic osteoarthritis. These can result in chronic pain, stiffness, and functional impairment [2].

One of the most important structures of the TMT joint is the Lisfranc ligament complex as it plays a significant role in stabilising and maintaining the midfoot arch [3]. The Lisfranc ligament complex comprises the first and second TMT ligaments, as well as the plantar interosseous ligament that attaches between the lateral aspect of the medial cuneiform and the medial aspect of the second metatarsal (Lisfranc ligament) [3]. Injury to the Lisfranc ligament complex is commonly referred to as a Lisfranc injury.

The manifestation of Lisfranc injuries can be varied. It ranges from sprains of the Lisfranc ligament complex to ligamentous disruption and even a combination of ligamentous injury with associated fractures. These injuries can be managed conservatively or with surgery. With advances in surgical techniques, indications for conservative management with cast immobilisation is limited [4]. The aim of surgery is to achieve anatomical reduction, stabilise the Lisfranc joint, and provide patients with the best chance of recovering with minimal functional limitation. Various techniques have been described for the surgical management of Lisfranc injuries. Theoretically, reduction and internal fixation (RIF), done open or percutaneously, is the ideal surgical technique to achieve stable anatomical reduction [5]. However, a significant number of patients who underwent RIF suffered from post-traumatic osteoarthritis (OA) and required conversion to arthrodesis. Implant-related complications necessitating a second surgery for hardware removal also occurred in more than 75% of the patients [6-8]. Primary arthrodesis was therefore proposed to be an alternative surgical modality. However, it has been reported that patients who underwent primary arthrodesis also experienced implant-related complications or nonunion, resulting in additional surgeries [9].

An alternative suspensory device fixation method using interosseous nonabsorbable sutures with endobuttons has been described in the literature with satisfactory results [10]. This technique can potentially overcome the implant-related complications associated with ORIF and primary arthrodesis.

The primary aim of this study is to explore patient-reported outcome measures (PROMs) after suspensory device fixation of Lisfranc injuries in a Southeast Asian urban population.

## Materials and methods

This was a single-centre retrospective study conducted in an adult tertiary hospital in Singapore with ethics approval obtained from a local institutional review board. Clinical records of patients who had a Lisfranc joint injury treated with the suspensory device fixation technique by the senior author between 2017 and 2019 were reviewed. Once patients were identified, they were contacted, and the nature of the study was explained to them. Data collection commenced once informed consent was obtained.

Data collected included basic demographic information, nature of the injury sustained, type of surgery performed, and pre- and post-injury levels of activity (daily living and sports). PROMs were evaluated with the aid of clinical questionnaires. The Foot and Ankle Outcome Score (FAOS) included domains of symptoms, pain, daily function, recreational function, and quality of life. The Foot and Ankle Ability Measure (FAAM) had two main subscales and patients answered questions related to function during activities of daily living and during sports. Each domain had a maximum score of 100 to indicate the best outcome. Questions of “Are you satisfied with the surgery?” and “would you undergo the surgery again?” were added to this survey.

Values of the FAOS and FAAM scores were reported in median with IQR (25th, 75th). The Mann-Whitney U test was performed to compare medians between groups with significance set at p <0.05.

**Figure 1:**
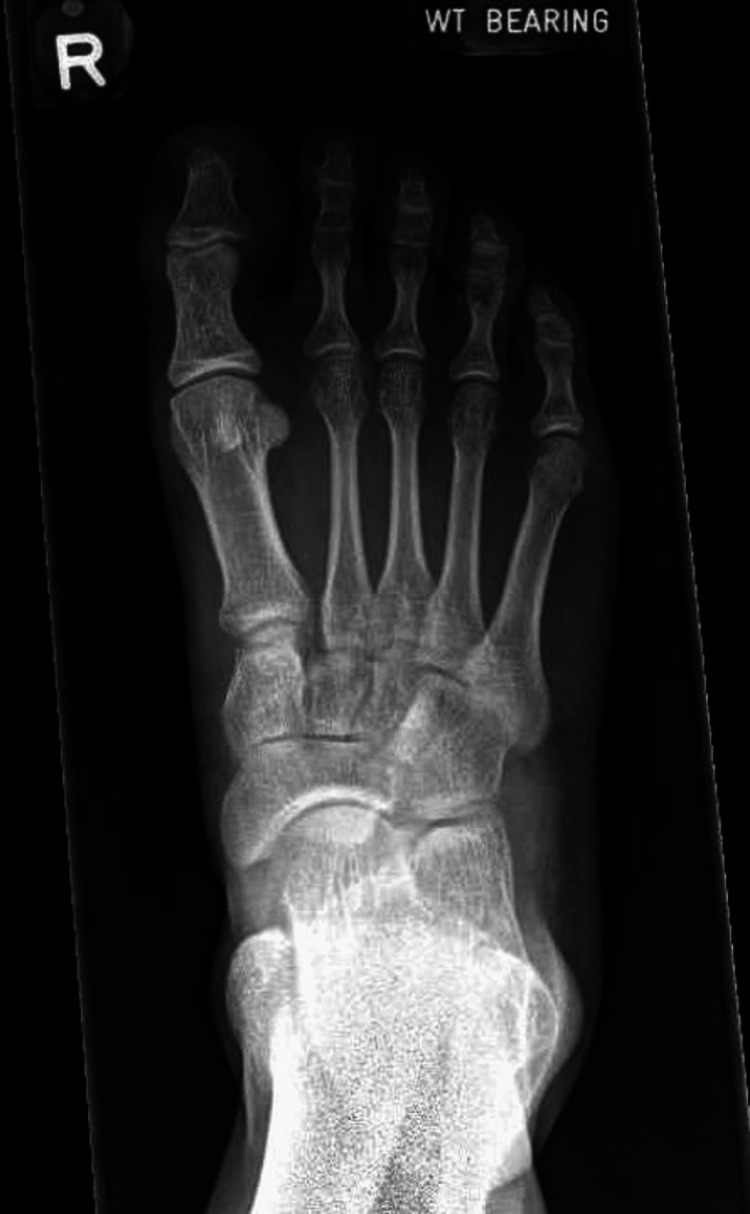
Pre-operative dorsoplantar view of the right foot demonstrating Lisfranc injury

**Figure 2:**
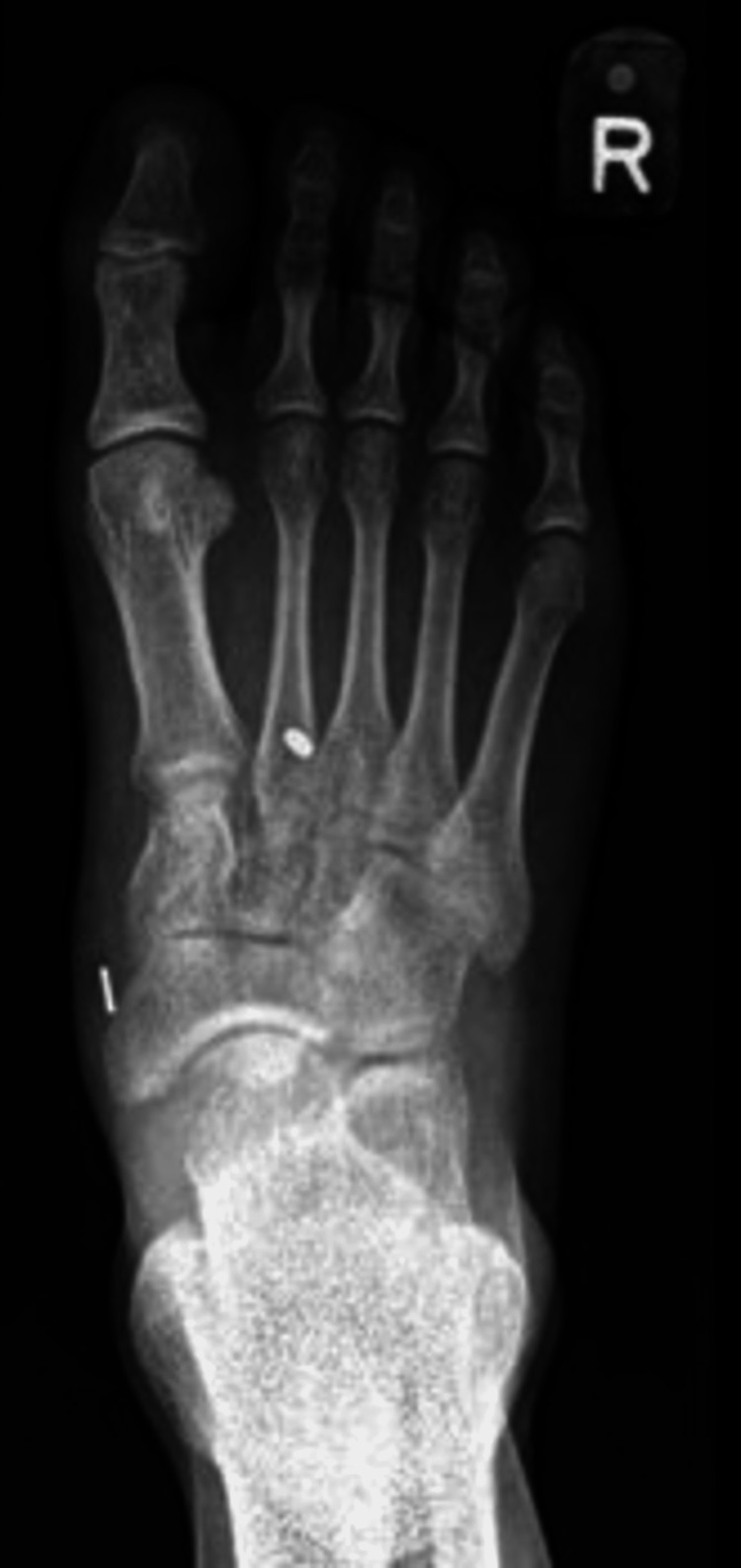
Postoperative dorsoplantar view of the right foot demonstrating Lisfranc injury fixed with a suspensory device

**Figure 3:**
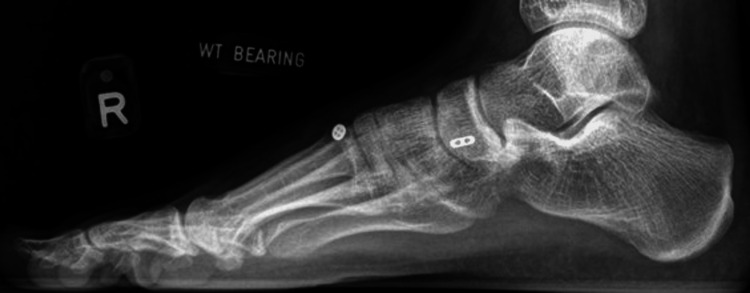
Postoperative lateral view of the right foot demonstrating Lisfranc injury fixed with a suspensory device

All surgeries were performed by a single foot and ankle surgeon with experience using the suspensory device technique a Mini TightRope® (Arthrex, Inc., Naples, FL). In this series, fixation would start from proximal to distal, medial to lateral. An incision was made over the Lisfranc joint. With direct visualisation of the joint, a reduction was achieved with a clamp. Following which, the Arthrex Mini Tightrope was used as the suspensory device, inserted from the second metatarsal to the medial cuneiform. Figure 1 presents a radiograph of a patient’s right foot with a Lisfranc injury. Figures 2-3 present the dorsoplantar and lateral radiographs after fixation with a suspensory device. In some cases, based on the senior author’s judgement of the patients’ injury morphology (e.g. other fractures), additional implants would be used.

Postoperatively, patients were kept non-weight-bearing on the affected limb for six weeks. Physiotherapy was commenced immediately post-operatively, and the patients were allowed the full range of motion of the ankle joint and toes. It was not routine to remove the endobuttons and interosseous suture. Patients who received internal fixation with screws and/or plates subsequently underwent hardware removal eight to twelve weeks postoperatively if they were agreeable to undergo a second surgery.

## Results

Twenty-nine patients gave informed consent for participation. Nineteen were male, and 10 were female. Sixteen underwent suture button fixation only, and 13 underwent suture button fixation in addition to the use of metal implants (intercuneiform screw fixation and/or plating). Patient ages ranged from 21 to 76 years with a mean age of 29 years old. Follow-up duration post-operatively was a minimum of one year. Sporting activities patients participated in varied activities from triathlon and high-impact sports such as rugby to low-intensity steady-state activities such as brisk walking in older patients. At the time of the interview, 28 patients (97%) were able to return to their pre-injury level of activity, and 27 patients (93%) were able to return to sports. All but one patient reported satisfaction with the outcomes of surgery. Four patients reported they would not wish to undergo surgery again because of the development of numbness or paresthesia post-operatively (Table 1). The median scores for the FAOS and FAAM questionnaires ranged from 81.25% to 100% across all domains (Table 2). 

**Table 1:** Values were presented as N = number of patients and percentage of total sample size (%)

Outcome	Yes	No
Return to pre-injury level of daily activity	28 (96.15)	1 (3.85)
Return to sports	27 (93.10)	2 (6.90)
“Are you satisfied with surgery?”	28 (96.15)	1 (3.85)
“Would you undergo the surgery again?”	25 (86.21)	4 (13.79)

**Table 2:** Total scores for FAOS and FAAM questionnaires at one year; values were presented in median (IQR)

Variables	N=29
Symptoms subscale	100 (92.86, 100)
Pain subscale	97.22 (94.44, 100)
Function subscale	100 (97.06, 100)
Sport subscale	90 (75, 100)
Quality of life	81.25 (68.75, 100)
Sports subscale current function level (%)	90 (80, 90)
Daily living subscale current level of function (%)	90 (80, 90)

Subgroup analysis was performed for the 13 patients who had suture button fixation in addition to the use of metal implants (SBM). The median scores on the FAOS and FAAM ranged from 87.5 to 100 for all domains (Table 3). There was no statistically significant difference when compared to the group of 16 patients who only had suture button fixation (SB).

**Table 3:** Comparison of scores between suture button only and suture button with additional metal implants

Patient-reported outcome domains	Suture button (n=16)	Suture button with additional metal implants (n=13)	P value
Symptoms subscale	98.2 (92.9, 100)	100 (92.9, 100)	0.392
Pain subscales	98.6 (92.4, 100)	97.2 (95.8, 100)	0.780
Function subscale	100 (96, 100)	98.5 (97.1, 100)	0.924
Sport function	90 (62.5, 100)	90 (80, 100)	0.561
Quality of life	81.3 (64.1, 93.8)	87.5 (65.6, 100)	0.609
Sport subscale current function level (%)	87.5 (76.3, 90)	90 (82.5, 92.5)	0.497
Daily living subscale current level of function (%)	85 (76.3, 90)	90 (82.5, 92.5)	0.406

No patients experienced surgical site infection, post-traumatic arthritis, or had hardware failure necessitating removal of the suspensory device at the time of follow-up.

## Discussion

Isolated ligamentous Lisfranc injuries may be easily missed [2]. These injuries commonly arise from low-energy trauma such as during sporting activities [11]. Conventional radiographs of the injured foot may not demonstrate widening between the bases of the first and second metatarsal. Therefore, weight-bearing dorsoplantar views comparing both feet can be considered in the diagnosis of subtle Lisfranc injuries [12]. Advanced imaging such as computed tomography (CT) scans and magnetic resonance imaging (MRI) may also be considered. Once the diagnosis of a Lisfranc injury has been made, the treating clinician has to consider the overall clinical status of the patient when deciding treatment. Given the advances in surgical techniques, the indication for conservative treatment is limited.

Anatomical reduction of the Lisfranc ligament complex has been demonstrated to be crucial in the recovery and function of the patient postoperatively [13]. A conventional option for the treatment of Lisfranc injuries is to achieve a reduction by fixing a screw through the medial cuneiform and into the base of the second metatarsal. However, this procedure is associated with several drawbacks. The use of rigid metal implants in the fixation of a ligamentous injury provides the patient with a non-physiological construct. The stiffness of the Lisfranc ligament has been reported to be 189.7 N/mm [14], while the stiffness of a screw is approximately 2240 N/mm [15]. The huge discrepancy in mechanical properties can lead to implant-related and ultimately functional issues. Screw-related complications have been reported to occur in up to 75% of cases [6-8]. Some surgeons practise routine removal of implants after 2 to 3 months to restore the elasticity of the midfoot [16]. However, this subjects patients to a second surgery, increasing overall operative risks, delaying the recovery, and contributing to further costs in the treatment of the injury. Interfragmentary screws can also damage the articular surface and result in arthritis [17]. To avoid cartilage damage, dorsal plate constructs may be used. However, this necessitates more extensive dissection, which leads to greater risks of infection and damage to neurovascular structures [18]. Similar to screw fixation, implant-related complications were also common among patients who had plating done [13]. The incidence of treatment failure among patients who underwent RIF and required revision surgery with arthrodesis has been reported to be up to 25% [7,18].

With the considerable number of patients requiring secondary fusion, primary arthrodesis was proposed as the index treatment of Lisfranc injuries [13]. This involves adequate joint preparation, reduction, and screw fixation between the medial cuneiform and base of the second metatarsal. Both open and arthroscopic joint preparation have been described [7,19]. However, screw-related complications requiring removal also occurred in a significant number of patients with incidence up to 19% [7,9]. Additionally, the loss of normal function in a young athletic patient after fusion is a significant detriment [20].

An alternative fixation of Lisfranc injuries with the use of a suspensory device was proposed [10]. The elasticity of a suture button construct has been reported to be 133 N/mm [15]. This is a lot more similar to that of the Lisfranc ligament (189.7 N/mm) in comparison to metal screws [14]. The physiologically analogous nature of the suture button construct also negates the need for routine removal. This can lead to a quicker recovery, providing higher levels of patient satisfaction. The elimination of a second surgery also reduces operative risk and cost for the patient.

When compared to the RIF, the suture button construct demonstrated superiority. Existing studies of RIF report 61%-83% of patients being able to return to pre-injury levels postoperatively [7,8]. In this study, 97% of patients achieved this outcome. Furthermore, when comparing the median scores for each FAOS and FAAM domain, values reported in this study were higher than previous RIF studies [7,8,21]. Compared with primary arthrodesis, the proportion of patients who were able to return to pre-injury activity levels was similar, with studies reporting rates of 86%-92% [7,8]. Nevertheless, it is worth noting that this study again demonstrated higher median scores for each FAOS and FAAM domain compared to primary arthrodesis [7,8].

In the subgroup analysis of patients with Lisfranc injuries and associated foot fractures requiring both the suture button construct and metal implants, patient-reported outcome measures were also satisfactory. The FAOS score was between 87.5 and 100 across all domains. Other studies involving patients with similar injury morphology have reported FAOS scores for primary arthrodesis and RIF of 58-80 and 81-86 across all domains, respectively [21]. When compared to the subgroup that only required suture button fixation, there was no significant difference in the results. This is an indication that in cases where there are concomitant ligamentous and bony injuries, the suture button construct can be used in conjunction with metal implants to achieve a good functional outcome.

There is no clear consensus regarding the optimal treatment of Lisfranc injuries. This is partly due to the small number of injuries resulting in difficulties when designing a study with sufficient power. This series of Lisfranc injuries treated with a suture button construct demonstrated superior PROMs when compared to RIF and primary arthrodesis. There were also no cases of failure of the suspensory device requiring removal. The results can be attributed to the similarity of the biomechanical properties between the Lisfranc ligament and the suspensory device. Therefore, we hypothesise that an ideal suspensory device is one with identical biomechanical properties to that of the Lisfranc ligament. It was reported by Hopkins et al. that the Arthrex InternalBrace suspensory device has a stiffness rated at 200 N/mm [15]. Using such a device may potentially result in better outcomes. Further studies have to be done to compare different suspensory devices to RIF and primary arthrodesis for the treatment of Lisfranc injuries.

This study possesses several notable strengths. It comprises a consecutive series conducted by a single surgeon within a single tertiary hospital and with a standardised postoperative recovery protocol. This eliminates potential variations that could compromise the validity of the results, enhancing the reliability and comparability of the outcomes. The potential confounding factors and the variable accuracy of data collection via a registry method were not present.

However, there are also limitations to this study. Firstly, this was a retrospective study, and potential factors that can influence the outcomes of interest were not fully within control. Secondly, the focus of this study was on PROMs. PROMs are subjective and, hence, unable to provide objective measures of the postoperative outcome. Finally, the PROMs were collected at least one year postoperatively, subjecting the results to recall bias.

## Conclusions

This study has demonstrated that treatment of Lisfranc injuries with a suspensory device construct resulted in good outcomes with 97% of patients being able to return to pre-injury activity levels and 93% of patients being able to return to sports. It may not be necessary to perform primary arthrodesis in uncomplicated Lisfranc injuries. This technique is also advantageous because implant removal is not routinely required due to the design and biomechanical properties of suspensory devices.
